# Farmacogenômica e Doença Cardiovascular: Onde Estamos e Para Onde Vamos

**DOI:** 10.36660/abc.20200151

**Published:** 2020-10-13

**Authors:** Ricardo Stein, Thaís Beuren, Luis Ramudo Cela, Filipe Ferrari

**Affiliations:** 1 Universidade Federal do Rio Grande do Sul Porto AlegreRS Brasil Universidade Federal do Rio Grande do Sul, Porto Alegre, RS - Brasil; 2 Universidade Federal do Rio Grande do Sul Hospital de Clínicas de Porto Alegre Programa em Pós-Graduação em Cardiologia e Ciências Cardiovasculares Porto AlegreRS Brasil Programa em Pós-Graduação em Cardiologia e Ciências Cardiovasculares - Universidade Federal do Rio Grande do Sul - Hospital de Clínicas de Porto Alegre, Porto Alegre, RS - Brasil; 3 Universidade da Corunha CorunhaGalicia Espanha Universidade da Corunha, Corunha, Galicia - Espanha

**Keywords:** Doenças Cardiovasculares, Genes, Hereditariedade, Genoma, Perfil Genético, Farmacogenética, Biotransformação, Tratamento Farmacológico/eventos adversos, Saúde Pública

## Abstract

A farmacogenômica (FGx) investiga a interação entre genes e medicamentos. Através da análise de regiões específicas do DNA, informações sobre o perfil de metabolização do paciente para um determinado fármaco podem ser descritas, assim como o perfil esperado de resposta ao tratamento. Objetivamente, esse tipo de teste pode ter impacto no tratamento de pacientes que não estão respondendo adequadamente a um determinado medicamento, seja pela ausência dos efeitos esperados ou em virtude do aparecimento de efeitos adversos. Neste cenário, o objetivo desta revisão é o de informar o cardiologista clínico sobre esta importante área do conhecimento e atualizá-lo sobre o tema, procurando preencher as lacunas no que diz respeito à relação custo-benefício da aplicação da FGx nas doenças cardiovasculares, além de fornecer informações para a implementação da terapia guiada pela FGx na prática clínica.

## Introdução, DNA e os Genes

A farmacogenômica (FGx) é a ciência que estuda a interação entre genes e medicamentos. A partir da análise de regiões específicas do DNA, é possível obter informações sobre, por exemplo, o perfil de metabolização do paciente para um determinado fármaco, bem como o perfil esperado de resposta ao tratamento. A FGx também visa diminuir a ocorrência de eventos adversos aos medicamentos (EAM).^1^^,^^2^ As inúmeras pesquisas nesta área têm focado na identificação de genes que predispõem às doenças, modulam respostas aos fármacos, e afetam a concentração e a ação desses medicamentos, além de se associarem a reações adversas.^3^ É frustrante saber que a eficácia de alguns tratamentos medicamentosos varia entre 25% a 80%, sendo que somente um terço dos pacientes expostos ao uso de diferentes tipos de fármacos obtém os benefícios terapêuticos desejados.^4^

Entre as causas da variação na resposta individual à mesma posologia de um fármaco, pode-se destacar a idade, os fatores genéticos e imunológicos, as enfermidades e a ocorrência de interações entre princípios ativos.^5^ A variabilidade genética pode alterar tanto a farmacodinâmica, ou seja, a relação entre a dose administrada e os efeitos produzidos, quanto a farmacocinética, que relaciona os eventos de absorção, distribuição, metabolismo e excreção da substância à sua concentração sistêmica.^1^

A ocorrência de EAM constitui um problema de saúde pública no mundo todo, pois aumenta significativamente o tempo de hospitalização, além de ter sido considerada como a quarta dentre as seis causas mais frequentes de morte nos Estados Unidos nos últimos 20 anos.^6^^,^^7^ Lá, por exemplo, mais de dois milhões de pessoas são hospitalizadas^8^ e pelo menos 55.000 morrem por ano em decorrência da não resposta ao tratamento ou de EAM.^9^ No Brasil, os dados ainda são escassos, e a FGx pode ser uma ferramenta de auxílio tanto no tratamento do paciente quanto na otimização dos gastos financeiros. Foi evidenciado que no Hospital de Clínicas de Porto Alegre, Brasil, 14% dos pacientes procuraram a emergência por EAM, e as classes de medicamentos mais envolvidas foram os antirretrovirais, os anticoagulantes, e os anti-hipertensivos. Daqueles que procuraram a emergência uma primeira vez, 20-30% retornaram. Os pesquisadores evidenciaram que o custo médio para tratar um paciente por EAM durante um ano foi de R$ 2200,00, sendo o custo total de 18 milhões de reais.^10^

Em uma publicação recente, foi realizada uma estimativa nacional de casos de morbimortalidade relacionados ao uso de medicamentos no Sistema Único de Saúde (SUS), utilizando dados do Datasus.^11^ A estimativa foi de que dos 150 milhões de brasileiros que vão ao médico pelo menos uma vez ao ano, 86% saem com prescrição de algum medicamento. Os danos causados por medicamentos, além de graves do ponto de vista clínico, custam bilhões ao ano para o SUS: a cada real investido no fornecimento de medicamentos, o governo gasta cinco reais para tratar as morbidades relacionadas a eles. As mais onerosas são as causadas por reações adversas (39,3% dos gastos), a não adesão ao tratamento (36,9%) e o uso de doses que não são as habitualmente recomendadas (16,9%). Metade dos casos poderia ser evitado com uma supervisão mais cuidadosa e efetiva dos diferentes tratamentos. Por fim, 60 bilhões por ano foi a estimativa de gastos no sistema de saúde público brasileiro com a morbimortalidade relacionada ao uso de medicamentos (30% do orçamento inicial do SUS).^11^

A doença cardiovascular (DCV) é a principal causa de morte no mundo, contribuindo significativamente para o crescente ônus econômico na área da saúde. Em 2016, 31% de todas as mortes no mundo (17,9 milhões) foram causadas por DCVs; aproximadamente 555 bilhões de dólares foram gastos nos Estados Unidos, e as previsões informam que esses custos aumentem para US$ 1,1 trilhão no ano 2035.^12^

Por essas razões, os exames de FGx, os quais são mais difundidos em países como Estados Unidos, Espanha e Canadá, vêm ganhando espaço no Brasil, tendo potencial de modificar para melhor a relação medicamento-médico-paciente. Com o auxílio da FGx, o médico poderá ter mais segurança e assertividade ao prescrever o medicamento adequado e a dose correta, já que possuirá informações importantes sobre o perfil genético do paciente, sem deixar de levar em conta outros fatores importantes relacionados ao indivíduo que está sendo tratado.^13^ Assim, a DCV está na vanguarda da terapia guiada por FGx, sendo interessante que os cardiologistas estejam atentos para as informações relativas a essa área do conhecimento.

Sabemos que existem várias classes de medicamentos para reduzir o risco de DCV, como também existe uma variação significativa na resposta ao tratamento.^14^ Além da variação que pode ser atribuída a diversas características sociodemográficas, há determinantes genéticos da droga, assim como respostas que podem afetar a maneira como as drogas são metabolizadas, absorvidas e distribuídas.^1^^,^^14^^-^^16^ Portanto, dados genéticos podem ser usados para identificação e avaliação das respostas a doses de drogas, controle de efeitos colaterais e também para previsão de resultados.^17^^-^^19^ Nos últimos anos, devido ao desenvolvimento na clonagem de genes, na genotipagem e no sequenciamento de DNA, a FGx emerge como um componente útil. O conhecimento atual pode ser aplicado em um gene individual, em uma área terapêutica ou em medicamento específico: a) uso de testes FGx para prever a dose individual de medicamento; b) uso de testes FGx para prever a ausência de resposta a um medicamento; e c) uso de testes FGx para prever indivíduos com sério risco de toxicidade se um medicamento for prescrito ou administrado.

Várias diretrizes clínicas nessa área do conhecimento estão disponíveis, sendo as principais delas: o *Clinical Pharmacogenetics Implementation Consortium* (CPIC),^20^ o *Dutch Pharmacogenetics Working Group* (DPWG),^21^ o *Canadian Pharmacogenomics Network for Drug Safety* (CPNDS),^22^ o *Groupe de Pharmacologie Clinique Oncologique* (GPCO/Unicancer),^23^ o *Réseau National de Pharmacogénétique Hospitalière* (RNPGx),^24^ e o *American College of Rheumatology* (ACR).^25^

### Um Pouco de História

Umas das primeiras terapias baseadas em uma mutação específica, que mudaram significativamente o prognóstico de doenças, são o trastuzumab no câncer de mama HER-2 positivo e o imatinibe na leucemia mieloide crônica.^26^^,^^27^ Desde então, a oncologia aposta progressivamente na utilização da informação genética, e atualmente esta serve para pautar a decisão terapêutica, tendo incluído o teste genômico em 39% dos ensaios clínicos de oncologia em 2018.^28^ Além da oncologia, inúmeras outras áreas identificaram ou aprimoraram tratamentos baseados em variações genéticas. Para a fibrose cística já foram identificadas mais de 100 mutações causadoras, o que, apesar de dificultar o desenvolvimento de tratamento específico para cada variante, possibilitou o agrupamento dos seus subtipos que parecem responder a tratamentos semelhantes.^29^

Os avanços da medicina genômica não se limitam apenas a drogas que agem a nível proteico. Técnicas como o CRISPR (*clustered regularly interspaced short palindromic repeats*) – uma região especializada do DNA – são usadas para silenciar genes e evitar o desenvolvimento de doenças em embriões e/ou modificar genes relacionados à doença em adultos.^30^ Essas técnicas são parte do que se conhece como terapia genética e, apesar de estarem em fase embrionária, são esperadas como alternativas potencialmente revolucionárias.

### Metabolização de Medicamentos

As principais diretrizes de FGx usam termos de consenso que visam facilitar a aplicação clínica dos resultados genéticos e harmonizar o relatório entre os diferentes laboratórios.^31^ Essa classificação é distinta para diferentes tipos de genes, e leva em consideração a combinação de variantes detectadas no mesmo gene e sua “zigosidade”. Um exemplo é a classificação consensual do citocromo P450 2D6 (CYP2D6), uma das principais enzimas do metabolismo de medicamentos, que está envolvida no metabolismo de aproximadamente 25% dos medicamentos comercializados. Os pacientes podem ser classificados em quatro fenótipos em relação ao seu perfil de metabolização dos medicamentos: a) metabolizadores lentos, b) metabolizadores intermediários, c) metabolizador rápidos, e d) metabolizadores ultrarrápidos, detalhados a seguir:

#### • Metabolizadores Lentos

Os pacientes apresentam uma quebra muito lenta dos medicamentos, tornando os efeitos colaterais mais pronunciados. Os indivíduos deste grupo geralmente são portadores de dois alelos com variantes que provocam redução, ou mesmo inatividade da enzima. Ainda, as doses padrão de certos medicamentos podem não funcionar como esperado. Até 15% da população se encaixa neste subgrupo.^32^

#### • Metabolizadores intermediários

Os metabolizadores intermediários podem de alguma forma afetar a quebra dos medicamentos, causando efeitos semelhantes aos metabolizadores lentos, mas não de forma tão pronunciada.^33^

#### • Metabolizadores rápidos

Estes indivíduos possuem uma taxa do metabolismo tida como “normal”. A medicação provavelmente funcionará conforme o planejado, e essas pessoas utilizarão as doses recomendadas na bula dos remédios.^34^

#### • Metabolizadores ultrarrápidos

Os pacientes desse grupo metabolizam os medicamentos muito rapidamente, pois possuem alelos que produzem enzimas com elevada atividade ou apresentam cópias extras de alelos (ex.: duplicações ou triplicações do gene).^35^

O gene CYP2D6, especificamente, é responsável pelo metabolismo em cerca de 25% dos medicamentos prescritos,^36^ e possui alelos que podem causar os quatro tipos de metabolismo descritos anteriormente.^37^ Estes alelos têm uma prevalência que varia de acordo com a etnia. Por exemplo, um dos principais alelos não funcionais conhecidos, o CYP2D6*4, tem prevalência estimada de 25% em indivíduos caucasianos; já o alelo CYP2D6*10 e CYP2D6*17 (ambos de função reduzida) são mais comuns em africanos e asiáticos, com uma frequência alélica de aproximadamente 40%.^38^

### Ensaios Clínicos Randomizados

Diversos estudos vêm sendo realizados nos últimos anos para testar o papel da FGx na prática clínica. Em um ensaio clínico randomizado (ECR),^39^ 1.956 pacientes infectados pelo vírus da imunodeficiência humana (HIV) foram divididos em dois grupos. O primeiro foi rastreado prospectivamente quanto à presença do alelo HLA-B*5701 e, aqueles positivos, não receberam o antiviral abacavir. O outro grupo recebeu tratamento padrão com abacavir sem rastreamento prospectivo quanto à presença de HLA-B*5701 (grupo controle). A incidência de hipersensibilidade ao medicamento foi menor no grupo que passou pelo rastreamento genético (3,4%) em comparação ao grupo controle (7,8%). Este resultado levou a *Food and Drug Administration* (FDA) dos Estados Unidos a incluir a exigência do teste FGx na bula do medicamento.^39^ Mais recentemente, o estudo de Smith et al.^40^ evidenciou redução de 30% na intensidade de dor em usuários crônicos de opioide quando a terapia era guiada pela presença da variante no gene CYP2D6.^40^ Uma meta-análise, incluindo cinco ECRs, encontrou 1,71 mais chance de remissão de sintomas em pacientes recebendo terapia guiada por genotipagem quando comparada à terapia usual.^41^

Focando na área de DCVs, os estudos mais numerosos são relacionados aos antiagregantes plaquetários e aos anticoagulantes. Após observações retrospectivas de que a presença de variantes genéticas classificadas como perda de função apresentava impacto nos efeitos do clopidogrel, iniciativas surgiram para avaliar o benefício de incluir o teste de forma rotineira. Investigadores da *Implementing Genomics in Practice* (IGNITE) evidenciaram em um grupo de 1.815 pacientes, maior taxa de eventos cardiovasculares naqueles com variantes de perda de função do gene CYP2C19 e em uso de clopidogrel, comparado com antiplaquetários alternativos, como o prasugrel ou o ticagrelor (hazard ratio [HR] 2,26, intervalo de confiança [IC] 95% 1,18-4,32; p=0,013).^42^ Outro ECR mostrou diminuição importante nos eventos coronarianos tardios com a implementação da estratégia FGx para uso do clopidogrel.^43^

Já em relação à varfarina, a maioria dos estudos avaliou variantes genéticas ligadas ao seu metabolismo nos genes CYP2C9 e VKORC1. O *European Pharmacogenetics of anticoagulant therapy* (EU-PACT) mostrou que, guiando a terapia pelo teste genético, houve aumento significativo no tempo em que os pacientes mantinham-se com um INR (*International Normalized Ratio*) dentro da faixa terapêutica (2,0-3,0).^44^ Mais recentemente, o estudo *Genetics Informatics Trial of Warfarin to Prevent Deep Vein Thrombosis* (GIFT) mostrou diminuição de sangramentos significativos, tromboembolismo venoso e morte no período perioperatório em pacientes com terapia guiada por FGx para cirurgias eletivas de implante de próteses de quadril e de joelho.^45^ Ambos os estudos citados relacionados à varfarina incluíram uma população majoritariamente branca e, por isso, há a necessidade de se incluir variantes do CYP2C9 mais comuns em populações de ascendência africana para obtenção de resultados mais acurados relacionados a esse grupo populacional. Por sua vez, o maior dos estudos da varfarina, o *Clarification of Optimal Anticoagulation Through Genetics* (COAG) mostrou-se diferente dos demais e relatou não haver diferença em se iniciar terapia com varfarina guiada por informação clínica ou guiada pela pesquisa de variantes do gene CYP2C9, as quais são muito mais comuns em população de ascendência europeia, em uma coorte composta apenas por 27% de afro-americanos.^46^ Por outro lado, e considerando o custo-benefício relacionado ao uso da varfarina e do clopidogrel, uma revisão sistemática recente que incluiu 31 ECRs mostrou que, comparado à terapia padrão, o teste FGx foi superior em 81% das vezes.^13^

Paralelamente aos estudos de medicamentos isolados, o conceito de teste preventivo vem crescendo e apresentando algumas evidências de benefício. Em 2012, Schildcrout et al.^47^ mostraram, em uma coorte de 52.942 pacientes em cuidado domiciliar, uma exposição de 64,8% a pelo menos um medicamento de mecanismo influenciado por variantes genéticas. Os autores estimaram em 398 o número de eventos adversos potenciais que poderiam ter sido evitados com a genotipagem preventiva. Já o estudo do genótipo de 44.000 participantes do biobanco da Estônia mostrou que 99,8% desses indivíduos tinham genótipo associado a um risco aumentado a pelo menos uma medicação.^48^ Com resultados concordantes, o protocolo RIGHT (*‘Right Drug, Right Dose, Right Tim eUsing Genomic Data to Individualize Treatment’*) criado pela clínica Mayo/iniciativa eMERGE, sequenciou painel incluindo os genes SLCO1B1 (membro da família do transportador de ânions orgânicos transportadores de soluto 1B1), CYP2C19, CYP2C9, VKORC1 e CYP2D6. Eles identificaram em 99% dos 1.013 indivíduos pelo menos uma variante de risco para uso de algum medicamento.^49^

Nos Estados Unidos, medicamentos com recomendações relacionadas à FGx perfazem 18% de todas as prescrições^50^ e 30% dos medicamentos com alto risco FGx mais prescritos representam 738 milhões de prescrições por ano.^51^ Tais informações vão à direção de que parece existir um impacto positivo do teste FGx preventivo, não apenas pelo aumento da eficácia terapêutica e custo-benefício, mas também pelo seu potencial de evitar EAMs. Além dos dados já mencionados, um estudo holandês também mostrou benefício pela redução de 73% para 28% no risco de na taxa de intoxicação por fluoropirimidinas em pacientes com dose guiada por genotipagem, além da redução das mortes induzidas pelo medicamento de 10% para 0%.^52^

### Importância da Farmacogenômica

Nos últimos anos a FGx surgiu como uma área de grande interesse e entusiasmo, pois lida fundamentalmente com a chamada “medicina personalizada”, levando em consideração a influência da variação genômica dos pacientes sobre suas respostas às medicações.^53^

Diferentes são os benefícios que podem ser alcançados com o advento da FGx, a citar:

- Aumento no poder da terapia e redução na probabilidade de intoxicação;
- Tratamentos iniciados no momento mais apropriado.

**Figura 1:**
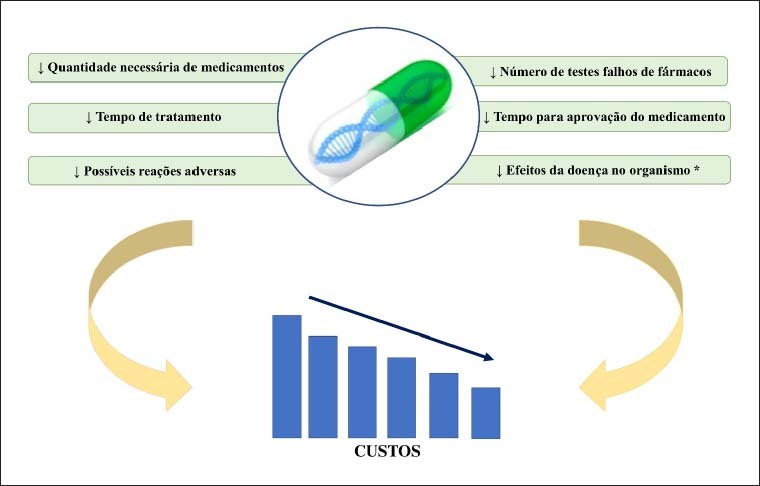
Vantagens da Farmacogenômica e Possível Redução nos Custos à Saúde. *Através da possibilidade de uma detecção mais precoce.

Além disso, a FGx pode contribuir para redução geral no custo dos cuidados de saúde, conforme apresentado na Figura 1. É importante mencionar que quase 200 mil mortes/ano na Europa são relacionadas aos EAM, com um custo de aproximadamente 80 bilhões de euros.^54^

Estima-se que uma boa parcela dos pacientes não apresenta respostas consideradas satisfatórias aos medicamentos.^55^^,^^56^ Nesse sentido, a FDA recomenda, por exemplo, que testes FGx sejam realizados antes da quimioterapia com a droga mercaptopurina – utilizada comumente para pacientes com alguns tipos de leucemia aguda.^57^ Tal recomendação baseia-se no fato de que, como esta droga pode causar efeitos colaterais graves e aumentar o risco de infecção a depender da variante genética do indivíduo, a terapia pode não surtir o efeito desejado.

O *Genomics and Targeted Therapy Group*, um braço do departamento de Farmacologia Clínica da FDA, tem como objetivo garantir a aplicação apropriada das estratégias FGx por meio das suas funções de revisão regulatória, pesquisa, desenvolvimento de diretrizes e educação profissional. Parte desse trabalho incluiu a criação de uma tabela agrupando as orientações farmacológicas das 161 drogas, que até o momento contém informações genômicas em seus rótulos. Sua última atualização traz números expressivos de biomarcadores associados a drogas em diversas áreas da medicina, inúmeras delas amplamente utilizadas na prática clínica^58^ (Tabela 1).

**Tabela 1:** Relação do número de biomarcadores genômicos descritos no rótulo de medicações em diversas especialidades médicas com base na tabela fornecida pelo *Food and Drug Administration*

Área	Biomarcadores
Oncologia	167
Infectologia	35
Psiquiatria	34
Neurologia	29
Hematologia	25
Anestesiologia	23
Cardiologia	22
Gastroenterologia	17
Reumatologia	11
Pneumologia	10
Endocrinologia	7
Erros inatos do metabolismo	7
Urologia	5
Dermatologia	4
Toxicologia	2
Transplante	1

Por fim, espera-se que, em breve, a FGx seja mais acessível e que a sua utilização consciente possa contribuir para que os médicos prescrevam as medicações com maior “acurácia” e os pacientes possam recebê-las com maior chance de sucesso terapêutico aliado ao menor risco de EAM.

### Associação entre Variantes Genéticas e Respostas aos Medicamentos na Doença Cardiovascular

**Figura 2:**
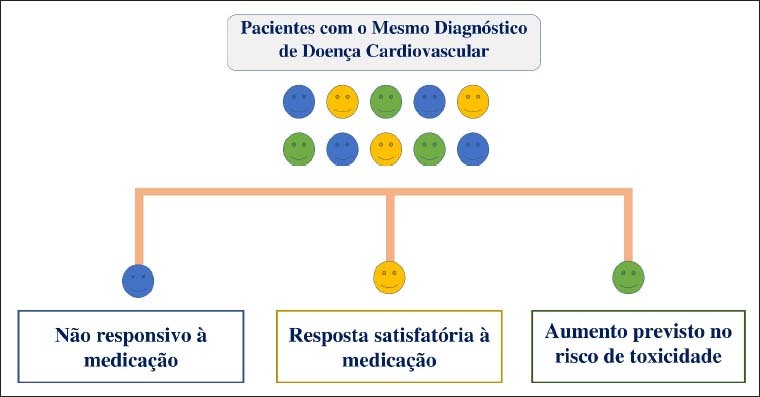
Potenciais Aplicações Clínicas da Farmacogenômica. Adaptado de Johnson, 2003.^99^

É de amplo conhecimento que fatores como idade, comorbidades, peso, bem como aqueles demográficos, podem contribuir para diferenças significativas nas respostas a uma mesma terapia farmacológica, bem como ao desenvolvimento de EAM.^59^^,^^60^ Nesse contexto, a variação genética pode representar um pilar fundamental para esse desfecho. Acredita-se que muitas mortes ao redor do mundo poderiam ter sido evitadas se o médico tivesse conhecimento prévio do perfil FGx dos pacientes, e pudesse empregar a medicação em doses corretas para aquele indivíduo.^61^ Pacientes com um mesmo diagnóstico (por exemplo, infarto agudo do miocárdio) são normalmente tratados da mesma maneira, embora suas respostas à terapia medicamentosa possam ser distintas. A “terapia sob medida” pode reduzir os EAMs e aumentar as taxas de eficácia, conforme ilustrado na Figura 2. Por exemplo, há uma enorme variação na dose diária necessária de um dos anticoagulantes mais utilizados na prática clínica, a varfarina, podendo variar em até 20 vezes.^62^ Por sua vez, o propranolol (betabloqueador) pode ter a sua dose variando em até 40 vezes a depender de qual paciente esteja recebendo a droga.^60^ Alguns fármacos utilizados em larga escala na prática clínica do cardiologista, os quais podem ter importantes associações genéticas, são apresentados na Tabela 2.

**Tabela 2:** Associação entre genes e medicamentos

Genes	Medicamentos	Classe	Variante gênica associada	Efeito associado ao alelo
*CYP2C9, VKORC1*	Varfarina	Antagonista da vitamina K	CYP2C9*2 (p.Arg144Cys; rs1799853)	Depuração reduzida de medicamentos; necessidade de dose reduzida
			CYP2C9*3 (p.Ile359Leu; rs1057910)	Depuração reduzida de medicamentos; necessidade de dose reduzida
			VKORC1 (-1639G>A; rs9923231)	↑ Sensibilidade a medicamentos; necessidade de dose reduzida
*CYP2C19*	Clopidogrel	Inibidor do receptor P2Y_12_	CYP2C19*2 (c.681G>A; rs4244285)	↑ Risco de eventos cardiovasculares; perda de função; menor efeito antiplaquetário.
			CYP2C19*17 (c.-806C>T; rs12248560)	↑ Sensibilidade à medicação; ganho de função; ↑ Risco de sangramento
*SLCO1B1*	Sinvastatina	Inibidor da HMG-CoA redutase	SLCO1B1*5 (p.Val174Ala; rs4149056)	↑ Risco de desenvolvimento miopatia ou rabdomiólise
*ADRB1*	Atenolol, metoprolol	Betabloqueador	ADRB1 (p.Ser49Gly; rs1801252)	Melhor controle da PA; ↑ FEVE
			ADRB1 (p.Arg389Gly; rs1801253)	
*CES1*	Dabigatrana	Anticoagulante de ação direta	CES1 (G143E, rs71647871)	↓ Metabolismo do medicamento e seus metabólitos
*ITGB3*	Aspirina	Antiagregante plaquetário	ITGB3 (Pl^A1/A2^ [T1565→C], rs5918)	↓ Efeito antiplaquetário

PA: Pressão arterial; FEVE: Fração de ejeção do ventrículo esquerdo; CYP2C9: Citocromo P450 2C9; VKORC1: Subunidade 1 do complexo Epóxido Redutase da Vitamina K; CYP2C19: P450 2C19; SLCO1B1: Membro da família do transportador de ânion orgânico transportador de soluto 1B1; CYP4F2: Citocromo P450, família 1, subfamília A, polipeptídeo 2; ADRB1: Adrenoceptor Beta 1; CYP11B2: Citocromo P450, família 11, subfamília B, polipeptídeo 2; FUT4: Fucosiltransferase 4; CES1: Carboxilesterase 1;ITGB3: Integrina beta-3.

#### Varfarina

A varfarina é um fármaco pertencente à classe dos antagonistas da vitamina K, e vem sendo utilizada em grande escala na prevenção de eventos trombóticos.^63^ Evidências sugerem que a resposta do indivíduo à varfarina, bem como a outros antagonistas da vitamina K, pode ser influenciada de forma importante por variações genéticas na enzima hepática do citocromo P450 (CYP2C9) e na Subunidade 1 do complexo Epóxido Redutase da Vitamina K (VKORC1), alvo para estes fármacos,^64^^,^^65^ além de polimorfismos no membro 2 da subfamília F e família 4 do citocromo P450 4 (CYP4F2).^66^ Foi demonstrado que variações nos alelos CYP2C9*2 e CYP2C9*3 diminuíram a atividade enzimática da CYP2C9 e inibiram o metabolismo anticoagulante,^67^ enquanto o polimorfismo VKORC1- 1639G>A parece ter influência na resposta farmacodinâmica aos antagonistas da vitamina K.^68^ Devido a essas observações, a FDA apontou a necessidade de informações FGx na bula da varfarina.

Na prática, os portadores heterozigotos dos alelos de função reduzida CYP2C9*2 ou CYP2C9*3 podem necessitar de uma dose reduzida de varfarina em cerca de 30% e 47%, respectivamente, enquanto os portadores homozigotos CYP2C9*3 podem necessitar reduções ainda maiores (~80%).^69^^-^^71^ Por sua vez, a variante -1639 G>A do gene VKORC1 parece reduzir a expressão de proteínas, o que teoricamente representa uma dose menor de manutenção de varfarina em comparação com os não portadores desta variante.^72^ Da mesma forma, combinações de algumas variantes associadas a um metabolismo extremo tornam mais difícil a obtenção de um INR terapêutico de maneira sistemática nesses pacientes.^73^ Nesse cenário, as diretrizes do CPIC recomendam considerar um anticoagulante oral de ação direta (por exemplo, edoxabana).^74^

#### Clopidogrel

Nos Estados Unidos, estima-se que mais de três milhões de indivíduos recebam anualmente prescrição de clopidogrel após a colocação de um *stent*.^75^ Ele é um fármaco tienopiridínico do grupo dos antiagregantes plaquetários.^76^ A resposta do indivíduo frente ao clopidogrel pode sofrer alteração, determinada pelo polimorfismo do CYP2C19.^77^

A variante de perda de função do CYP2C19*2 foi associada a um risco aumentado de eventos cardiovasculares adversos, incluindo trombose de *stent* durante o tratamento com clopidogrel.^78^ Mais especificamente, o alelo CYP2C19*2 (rs4244285) causa perda de função e foi associado à redução na atividade antiagregante do medicamento.^79^ Além disso, portadores do alelo CYP2C19 *3 (rs4986893) também têm respostas reduzidas ao clopidogrel e uma taxa mais alta de eventos cardiovasculares adversos recorrentes em comparação com não portadores.^80^^,^^81^ É importante mencionar que as frequências dos alelos CYP2C19 *2 e CYP2C19 *3 são mais altas nas populações asiáticas, sugerindo que estes indivíduos têm maior probabilidade de serem resistentes à terapia com esta droga.^82^ Em contraste, o alelo CYP2C19*17 (rs3758581) promove ganho de função e tem sido associado ao aumento da atividade enzimática e à melhor inibição das plaquetas. Os portadores da variante CYP2C19*17 foram denominados metabolizadores ultrarrápidos.^83^

A raça parece ser outro fator com papel importante nesse cenário. Cresci et al.^84^ compararam o efeito do polimorfismo do CYP2C19 sobre eventos CVs adversos entre pacientes com infarto agudo do miocárdio em caucasianos e afro-americanos tratados com clopidogrel. Foi observado que o alelo CYP2C19*2 teve associação significativa com aumento da taxa de mortalidade em um ano e a uma tendência no aumento na incidência de infarto do miocárdio recorrente em caucasianos. Já o alelo CYP2C19*17 foi associado a um aumento na mortalidade em um ano e a um risco aumentado de sangramento em afro-americanos. É importante mencionar que pacientes submetidos à intervenção coronária percutânea que possuem pelo menos um alelo CYP2C19*2 podem apresentar maior risco de trombose de *stent*. Em um ECR,^85^ aproximadamente 2.500 pacientes foram pré-tratados uniformemente com 600 mg de clopidogrel. Os portadores do alelo CYP2C19*2 apresentaram um aumento significativamente maior na incidência de trombose de *stent* em 30 dias, quando comparados com os portadores do alelo CYP2C19 de tipo selvagem.^85^ Nessa mesma linha, a meta-análise conduzida por Mega et al.,^86^ que arrolou estudos com pacientes mais complexos e em tratamento agressivo, encontrou um risco aumentado de trombose de *stent* quando o alelo *2 foi identificado pela FGx.

Apesar dessas evidências, uma revisão sistemática com meta-análise abrangendo 15 estudos não corroboraram com tais achados, não indicando uma influência clara dos polimorfismos do gene CYP2C19 na eficácia clínica do clopidogrel,^87^ sugerindo que o uso de regimes antiplaquetários individualizados guiados pelo genótipo CYP2C19 não devam ser realizados.

Na atualidade, o *American College of Cardiology* em conjunto com a *American Heart Association* também não recomenda testes de FGx de rotina para o CYP2C19.^88^ No entanto, outra meta-análise mais recente demonstrou que os pacientes que se beneficiam do estudo FGx são aqueles com doença arterial coronária que são submetidos a procedimentos de revascularização miocárdica percutânea.^89^ Nesse contexto, o CPIC recomenda formalmente que pacientes com síndrome coronária aguda, ou ainda, aqueles submetidos à intervenção coronária percutânea, sejam submetidos ao teste FGx. O CPIC enfatiza que aqueles que possuem uma ou duas cópias do alelo com perda de função devem receber agentes antiplaquetários alternativos (como prasugrel ou ticagrelor), a fim de reduzir o risco de eventos cardiovasculares adversos.^90^ Por outro lado, outras populações de pacientes (por exemplo, fibrilação atrial), nas quais o uso do clopidogrel é mais discutível, o painel FGx não está indicado.

Claassens et al.^91^ realizaram um ECR recente para avaliar os resultados da terapia antiplaquetária guiada pelo genótipo CYP2C19 em pacientes com infarto agudo do miocárdio com supradesnível do segmento ST. Os pacientes foram alocados para receberem tratamento guiado por genótipo – pacientes sem variantes de perda da função CYP2C19 receberam clopidogrel e pacientes com variantes receberem terapia padrão (prasugrel ou ticagrelor). Não foi observada nenhuma diferença entre os grupos quanto à incidência de eventos trombóticos. Dessa forma, uma estratégia guiada por FGx foi não inferior à abordagem descrita como padrão (prasugrel ou ticagrelor), a qual é muito mais cara e que apresentou maior incidência de sangramento.^91^

Recentemente, os resultados do ECR TAILOR PCI foram apresentados. Este estudo avaliou uma estratégia guiada por genótipo (n = 2.652) *versus* terapia padrão (n = 2.650) em pacientes com doença arterial coronariana estável ou instável submetidos à intervenção coronária percutânea com objetivo de orientar a terapia antiplaquetária. No grupo randomizado para estratégia guiada por genótipo, os pacientes submetidos à genotipagem receberam ticagrelor 90 mg duas vezes ao dia (portadores de um alelo CYP2C19 *2 ou *3) ou clopidogrel 75 mg diariamente. No grupo denominado terapia padrão, os sujeitos receberam 75 mg de clopidogrel diariamente e passaram por genotipagem somente após 12 meses. O desfecho primário era composto por morte cardiovascular, infarto do miocárdio, acidente vascular cerebral, trombose de stent ou isquemia recorrente em 12 meses. O desfecho primário e a incidência de sangramento não foram diferentes entre os grupos de tratamento. Entretanto, cabe ressaltar a redução de 34% nesses eventos em um ano, bem como uma redução significativa de 40% no número total de eventos por paciente no braço guiado por FGx. Por fim, uma análise post hoc encontrou uma redução de aproximadamente 80% na taxa de eventos adversos nos primeiros três meses de tratamento nos pacientes randomizados para terapia guiada por genótipo.^92^^,^^93^

#### Betabloqueadores

Essa classe de drogas é amplamente utilizada para o tratamento de arritmias cardíacas, angina, infarto do miocárdio e hipertensão.^94^ Os genes associados à resposta inter individual do betabloqueador incluem o CYP2D6, o adrenoceptor beta 1 (ADBR1), o adrenoceptor beta 2 (ADBR2) e o receptor quinase 5 acoplado à proteína G (GRK5).^95^ Por exemplo, alguns betabloqueadores, incluindo propranolol e o metoprolol, são metabolizados pelo CYP2D6, e a perda de função desta variante é muito comum.^34^ Por sua vez, evidências sugeriram que pacientes hipertensos portadores do alelo Arg389 tipo selvagem homozigoto obtiveram três vezes maior redução da pressão arterial diastólica diurna com o uso do metoprolol quando comparados aos portadores do alelo Gly389.^96^ Embora com resultados ainda não tão consistentes, pacientes que são homozigotos do haplótipo Arg389 do ADBR1 parecem apresentar uma resposta mais satisfatória a toda a família dos betabloqueadores, apresentando melhor fração de ejeção do ventrículo esquerdo quando comparados aos portadores do alelo Gly389.^97^

Em relação à cor da pele, a frequência mais alta do alelo Gly389 em afroamericanos em comparação com os brancos pode ser uma explicação plausível para sua resposta reduzida aos betabloqueadores. Embora etnia e polimorfismos da ADRB1 tenham sido relatados como preditores independentes de resposta ao betabloqueador,^98^ outros estudos prospectivos elucidando os papéis dessas variantes genéticas na respostas étnico-específicas são justificados.

Assim, no cenário da insuficiência cardíaca, ainda não há recomendações para o uso de informações FGx que visem orientar o uso de betabloqueadores.

#### Estatinas

As estatinas são uma classe de fármacos que têm como alvo a inibição da 3- hidroxi-3-methyl-glutaril-CoA redutase (HMG-CoA redutase). Elas visam reduzir os níveis sanguíneos de colesterol, especialmente o LDL.^99^ Em conjunto com mudanças no estilo de vida, as estatinas são consideradas como terapia de primeira linha para prevenção primária e, especialmente secundária, de DCVs. Esses fármacos, por outro lado, apresentam uma ampla variabilidade interindividual na extensão da redução do LDL, explicada, em parte, por fatores ambientais e genômicos.^100^ Dessa forma, para que haja uma resposta mais eficaz, pode ser necessário um ajuste de dosagem para cada indivíduo.

Recentemente, Licito et al.^101^ avaliaram o perfil FGx relativo à dor neuromuscular em 76 pacientes portadores de diabetes tipo 2 e DCV prévia que estivessem em uso de fármacos antidiabéticos e anticolesterolêmicos, como a estatina. Foram estudadas diferentes variantes, tais como: SLCO1B1, ABCB1, ABCC8 e biotransformadores de drogas da família citocromo P450 (CYP), incluindo CYP2C9*2, CYP2C9*3, CYP2C8*3 e CYP3A4*22. Dos 35 pacientes tratados com estatinas, aproximadamente 17% apresentaram dor neuromuscular. A análise FGx mostrou ausência de correlação entre polimorfismos de genes candidatos e toxicidade, exceto para o alelo SLCO1B1 T521C. Assim, quando disponível, sugere-se a análise da variante SLCO1B1 T521C, permitindo que os médicos otimizem o tratamento prescrito, visando minimizar a dor neuromuscular e maximizar os benefícios da estatina.

Ainda, a variante mais significativamente associada ao gene SLCO1B1, c.521T>C, leva à diminuição da função de transporte do SLCO1B1, a qual pode reduzir a depuração das estatinas e aumentar chance de toxicidade do músculo esquelético. Uma meta-análise de nove estudos de caso-controle, englobando quase 4.500 pacientes, mostrou que indivíduos com o alelo variante C eram significativamente mais propensos a sofrer miopatia relacionada à estatina (CT + CC versus TT: odds ratio = 2,09; IC95% = 1,27-3,43).^102^

### Possíveis Barreiras à Implementação da Farmacogenômica

Graças ao avanço da tecnologia e das técnicas de sequenciamento, o custo da avaliação FGx diminuiu significativamente nos últimos anos (Lei de Moore), facilitando seu uso na prática clínica; porém, o custo relativamente elevado ainda representa uma barreira para a implementação mais ampla dessa ferramenta. Além disso, uma possível falta de familiaridade dos profissionais da saúde, a ausência de uma plataforma padronizando a investigação e o pensamento acadêmico e, de uma forma geral, o fato de que o volume de estudos que demonstram os benefícios da FGx ainda é insuficiente, são fatores que contribuem para uma baixa aceitação por parte da comunidade científica da inclusão de testes FGx na prática clínica.

No entanto, esforços para contornar esses obstáculos ocorrem atualmente de forma global e contam com estudos de grandes proporções,^103^ tais como, o *UK’s 100.000 Genomes Project*^104^ e o *PREemptive Pharmacogenomic testing for prevention of Adverse drug REactions* (PREPARE),^105^ o qual conta com a participação de sete países na Europa. No outro lado do Atlântico Norte, nos Estados Unidos, o *Electronic Medical Records and Genomics* (eMERGE),^106^ o *Network and the Implementation of Genomics in Practice* (IGNITE),^107^ e o *Clinical Sequencing Evidence Generating Research Consortium*,^108^ são parte de uma série de projetos custeados pelo *National Human Genome Research Institute*, sendo estimado um investimento na pesquisa genética de pelo menos US$ 775 milhões no período de 2007-2022. Na Ásia, o programa *South East Asian Pharmacogenomics Research Network* (SEAPharm) inclui cinco países para conduzir estudos de FGx.^109^ De forma geral, estes estudos visam definir, gerar e analisar evidências sobre a utilidade clínica do sequenciamento genômico para guiar terapia, custo-eficácia e o valor de sua implementação de forma vasta na prática médica. Dos resultados já obtidos, uma revisão que incluiu 44 avaliações de custo-benefício, mostrou que 30% delas mostrou custo-efetividade e 27% evidenciou até redução de custos,^110^ o que contribui para uma perspectiva otimista para o futuro da FGx.

Entre inúmeras contribuições das iniciativas globais para a expansão da FGx na prática clínica, vale destacar um de seus aspectos cruciais: a educação e o treinamento dos profissionais de saúde e a promoção e estímulo ao investimento em tecnologia nos centros de pesquisa e assistência. Além da contribuição com a literatura, os projetos promovem o treinamento de pessoas para a prática adequada da medicina genômica, a qual exige a adoção de atitudes básicas que talvez sejam menos relevantes em outras especialidades. Exemplos são a inclusão da família do paciente no aconselhamento e no plano de tratamento, além da confidencialidade que assegura o uso de informações genéticas com propósitos exclusivamente assistenciais (evitando seu uso inapropriado para imposição da lei, seguros, marketing ou relação empregatícia). A educação e treinamento têm papel central para a aceitação da medicina genômica na prática clínica, e a aceitação por sua vez é ponto chave para sua implementação. O esforço para geração de dados contribui significativamente, mas a disposição e engajamento dos profissionais da área da saúde que almejam a excelência da assistência médica são indispensáveis. A dimensão e o progresso dos esforços e investimentos que ocorrem globalmente deixam clara a relevância e o potencial atribuídos à medicina genômica, que já pode ser considerada parte da prática médica de alta qualidade e um dos pilares da medicina de precisão.

## Considerações Finais

1) Áreas de consenso: o teste FGx pode ser útil para o uso otimizado de diversos medicamentos, permitindo uma maior segurança farmacológica;
2) Áreas de controvérsia: se os testes FGx devem ser aplicados mais amplamente, inclusive na prescrição de certos medicamentos em que o benefício geral é menos claro, permanece controverso;
3) Área de crescimento: cada vez mais, as informações pessoais sobre genótipos são disponibilizadas diretamente ao consumidor. Isso provavelmente aumentará a demanda por prescrição personalizada, significando que os prescritores precisam levar em consideração as informações FGx. Como exemplo, podemos citar o Projeto 100.000 genomas. Este impressionante projeto fornecerá sequências genômicas completas que podem fazer parte de um prontuário médico do paciente. Esta informação parece ser de grande valor na prescrição personalizada;
4) Áreas oportunas para o desenvolvimento de pesquisas: desenvolvimento de novos medicamentos visando fatores de risco genéticos específicos para doenças. Estes podem ser prescritos para aqueles com genótipo de risco. É provável que as informações FGx estejam disponíveis rotineiramente no futuro, especialmente em ambientes tecnologicamente avançados. Isso poderia influenciar a prescrição de uma variedade de medicamentos além daqueles para os quais, atualmente, são necessários testes antes da prescrição.
