# Supplementary material for: Farmacogenômica e Doença Cardiovascular: Onde Estamos e Para Onde Vamos
Source: Arq Bras Cardiol. 2020 Oct 13;115(4):690–700. [Article in Portuguese] doi: 10.36660/abc.20200151 (PMC8386961; doi:10.36660/abc.20200151)
Supplement: Translation

# Translation: Pharmacogenomics and Cardiovascular Disease: Where are We and Where do We go from Here?

**Authors:** Ricardo Stein, Thaís Beuren, Luis Ramudo Cela, Filipe Ferrari

**Affiliations:** 1 Porto AlegreRS Brazil Universidade Federal do Rio Grande do Sul, Porto Alegre, RS - Brazil; 2 Porto AlegreRS Brazil Graduate Program in Cardiology and Cardiovascular Sciences - Universidade Federal do Rio Grande do Sul - Hospital de Clínicas de Porto Alegre, Porto Alegre, RS - Brazil; 3 La CoruñaGalícia Spain Universidad de Coruña, La Coruña, Galícia - Spain

**Keywords:** Cardiovascular Diseases, Genes, Heredity, Genome, Genetic Profile, Pharmacogenetics, Biotransformation, Drug Therapy/adverse effects, Public Health

## Introduction, the DNA and the Genes

Pharmacogenomics (PGx) is the science of understanding the interaction between genes and drugs. The analysis of specific areas of the DNA provides information about a certain drug metabolism and about the expected response to a certain treatment. PGx also aims to reduce the incidence of adverse drug events (ADEs).^1^^,^^2^ Many studies in this area have focused on the identification of genes that predispose to diseases, modulate drug response, affect drug concentration and correlate with adverse effects of patients exposed to different types of drugs, so the desired therapeutic benefit is achieved.^4^

The causes of individual responses to a same drug dosage include age, genetic and immunological factors, comorbidities and interaction between active principles.^5^ Genetic variability may influence not only pharmacodynamics, but also pharmacokinetics, which studies the relationship of the absorption, metabolism and excretion of the drug to its systemic concentration.^1^

ADEs are a public health problem in the world, as they significantly increase the length of hospital stay, and are considered the fourth of the six main causes of death in the United States in the last 20 years.^6^^,^^7^ Also, in the United States, more than two million people are hospitalized^8^ and at least 55,000 people die each year from abscence of treatment response or from ADE per se. In Brazil, data is still scarce and PGx could be a useful tool in the treatment of patients and optimization of financial expenses.^10^ The most common drug classes were antiretrovirals, anticoagulants and antihypertensives. In this study,^10^ the mean cost to treat a patient for ADE was BRL2,200, with a total cost of 18 million Brazilian reals.

In a recent publication, a national estimate of drug-related morbidity and mortality was conducted by the Brazilian Unified Health System (SUS) using data from the Datasus database.^11^ The estimate showed that of 150 million Brazilians that go to the doctor at least once a year, 86% leave the doctor with a drug prescription. The adverse events caused by the drugs are severe, not only from a clinical point of view, but also from an economical perspective – for every Brazilian real spent on drug provision, five Brazilian reals are spent on the treatment of drug-related comorbidities. The most expensive events are those caused by adverse reactions (39.3% of the expenses), non-adherence to treatment (36.9% of the expenses) and unusual dose regimens (16.9% of the expenses). Half of the cases could be prevented by a more careful and effective supervision of different therapies. Finally, it was estimated that 60 billion Brazilian reals were spent annually on drug-related morbidity and mortality by the SUS, corresponding to 30% of its initial budget.^11^

Cardiovascular disease (CVD) is the main cause of death in the world, significantly contributing to the increasing economic burden of health costs. In 2016, 31% of all deaths in the world (17.9 million) were caused by CVDs. These diseases cost the United States approximately $555 billion in that year, and estimates say that this cost will reach $1.1 trillion in 2035.^12^

For this reason, the use of PGx tests, which is more widespread in countries like the United States, Spain and Canada, has gained importance in Brazil, with the potential to improve the drug-physician-patient relationship. With the aid of PGx, the physician could prescribe, in a safer and more assertive way, the most appropriate drug at the correct dose, since, in addition to other important factors, information about patient’s genetic profile would be now available.^13^ Thus, CVDs are at the forefront of PGx-guided therapy, and cardiologists should be alert to this area of knowledge.

Several classes of drugs are known to reduce the risk of CVDs, but it is also known that there is a significant individual variation in treatment response.^14^ In addition to variations attributable to sociodemographic characteristics, there are genetic determinants of drug and responses that may affect how the drugs are metabolized, absorbed and distributed.^1^^,^^14^^-^^16^ Therefore, genetic data can be used in the identification and evaluation of drug response, control of side effects, and prediction of results.^17^^-^^19^ With recent advances in gene cloning, genotyping and DNA sequencing, PGx has emerged as a useful component. Current knowledge may be applied at an individual gene level, to a therapeutic area or to specific drug: (a) PGx tests to predict an individual dose of the drug; (b) PGx tests to predict individual risk of drug toxicity in response to a drug prescribed or administered.

There are many clinical guidelines available in this area of knowledge, and the most relevant ones are: the Clinical Pharmacogenetics Implementation Consortium (CPIC),^20^ the Dutch Pharmacogenetics Working Group (DPWG),^21^ the Canadian PGxs Network for Drug Safety (CPNDS),^22^ the Groupe de Pharmacologie Clinique Oncologique (GPCO/Unicancer),^23^ the Réseau National de Pharmacogénétique Hospitalière (RNPGx),^24^ and the American College of Rheumatology (ACR).^25^

### A Little of History

Some of the main therapies based on a specific mutation, that changed significantly the prognosis of diseases, are trastuzumab therapy against HER2-positive breast cancer and imatinib in chronic myeloid therapy.^26^^,^^27^ Since then, Oncology has bet on the use of genetic information and today it serves to guide the therapeutic decision making, having included the genomic test in 39% of the clinical trials in the field in 2018.^28^ In addition to oncology, other areas have identified and improved therapies based on genetic variations. For cystic fibrosis, more than 100 causative mutations have been identified, that, even though make the development of a specific treatment for each variant difficult, enables the grouping of subtypes that seem to respond to similar treatments.^29^

Advances in genomic medicine are not limited to drugs that act at the protein level. Techniques like the clustered regularly interspaced short palindromic repeats (CRISPR) system, which refers to a specialized DNA region, have been used to silence genes and prevent the development of diseases in embryos and/or modify disease-related genes in adults.^30^ These techniques are part of what is known as genetic therapy and, although at embryonic stage, they are expected as potentially revolutionary alternatives.

### Drug Metabolization

The main PGx guidelines have adopted terms that aim to facilitate clinical application of genetic results and harmonize reports from different laboratories.^31^ This classification varies with different types of genes, and takes into account the combination of variants identified in the same gene and its zygosity. One example is the consensual classification of cytochrome P450 2D6 (CYP2D6), one of the main enzymes of drug metabolism, which is involved in the metabolism of approximately 25% of the commercialized drugs. Patients may be classified into one of four phenotypes regarding the type of drug metabolizers: poor metabolizers, intermediate metabolizers, extensive metabolizers and ultrarapid metabolizers, as detailed below:

#### • Slow Metabolizers

Patients experience a very slow breakdown of medications, making side effects more pronounced. Patients of this group have two alleles with variants that cause a reduction, or even inactivity of the enzyme. Also, standard doses of certain drugs may not work as expected. Up to 15% of the population are in this group.^32^

#### • Intermediate Metabolizers

Intermediate metabolizers may somehow affect the breakdown of medications, causing effects similar to those in poor metabolizers, but not as pronounced.^33^

#### • Extensive Metabolizers

The rate of metabolism of these patients is considered “normal”. Medication is likely to work as planned, and these individuals will take the dose recommended in the package insert of the medication;^34^

#### • Ultrarapid Metabolizers

Patients in this group metabolize medications very quickly, due to the presence of two alleles that produce highly active enzymes or of extra copies of the alleles (e.g. gene duplication or triplication).^35^

The CYP2D6 gene, responsible for the metabolism of nearly 25% of the medications prescribed,^36^ has the alleles that generate the four metabolizer types, previously described.^37^ The prevalence of these alleles varies with ethnicity. For example, one of the main known nonfunctional alleles, the CYP2D6*4, has an estimated prevalence of 25% among Caucasians, whereas the CYP2D6*10 and the CYP2D6*17 (both with reduced function) are more common in African and Asian populations, with an allele frequency of about 40%.^38^

### Randomized Clinical Trials

Over the last years, several studies have been conducted to test the role of PGx in clinical practice. A randomized clinical trial (RCT)^39^ involving 1,956 patients infected with human immunodeficiency virus were randomly assigned to one of two groups – to undergo prospective HLA-B*5701 screening, with exclusion of HLA-B*5701-positive patients from abacavir treatment, or to undergo a standard-of-care approach of abacavir use without prospective HLA-B*5701 screening (control group). The incidence of hypersensitivity reaction was lower in the prospective-screening group (3.4%) than in the control group (7.8%). This result made the US Food and Drug Administration (FDA) require the inclusion of PGx test in the package insert of the medication.^39^ More recently, Smith et al.^40^ reported a reduction by 30% in pain intensity in chronic opioid users when the therapy was guided by the presence of CYP2D6. A meta-analysis including five RCTs found that individuals receiving genotyping-guided therapy were 1.71 times more likely to achieve symptom remission compared with individuals who received standard treatment.^41^

Regarding studies on CVDs, most are related to antiplatelet and anticoagulation agents. After retrospective observations that the presence of genetic variants classified as loss of function had an impact on the effects of clopidogrel, initiatives have emerged to evaluate the benefits of including PGx tests as a routine approach. The Implementing Genomics in Practice (IGNITE) investigators observed, in a group of 1,815 patients, higher rates of cardiovascular events in patients with a CYP2C19 loss-of-function allele prescribed clopidogrel compared with alternative antiplatelet therapy, including prasugrel or ticagrelor (hazard ratio [HR] 2.26, 95% confidence interval [CI] 1.18-4.32; p=0.013).^42^ Another RCT showed an important decrease in late coronary events with implementation of PGx strategy for clopidogrel prescription.^43^

Regarding warfarin, most studies evaluated genetic variants related to its metabolization in CYP2C9 and VKORC1 genes. The European Pharmacogenetics of anticoagulant therapy (EU-PACT) showed that a genotype-guided therapy significantly increased the percentage of time in the therapeutic range of 2.0 to 3.0 for the international normalized ratio (INR).^44^ More recently, the Genetics Informatics Trial of Warfarin to Prevent Deep Vein Thrombosis (GIFT) study showed a significant reduction in major bleeding, venous thromboembolism and death in patients on genotype-guided therapy with warfarin during the perioperative period of elective surgeries of hip or knee arthroplasty.^45^ These two studies involving warfarin included a predominantly white populations and therefore, further studies including CYP2C9 variants that are more common African-descendant populations are needed to obtain in accurate results related to these groups. Interestingly, the largest study on warfarin, the Clarification of Optimal Anticoagulation Through Genetics (COAG) study, showed contrasting results, reporting no difference in initiating warfarin therapy based on clinical information and initiating warfarin therapy based on individual’s genotype (search for CYP2C9 variants, which are far more common in European-descendant populations, in a cohort composed of 27% of African-Americans).^46^ On the other hand, considering the cost-benefit of the use of warfarin and clopidogrel, a recent systematic review including 31 RCTs showed that, the PGx test was superior to standard therapy in 81% of the times.^13^

Parallel to studies on one type of medication, the concept of preventive test has gained importance and shown evidence of benefit. In 2012, Schildcrout et al.^47^ estimated that 64.8% of 52,942 medical home individuals were exposed to at least one medication with a mechanism influenced by genetic variants. The authors also estimated that 398 potential adverse events could have been prevented with an effective preemptive genotyping. The study on genotype data of 44,000 participants of the Estonian Biobank showed that 99.8% of these individuals had a genotype associated with increased risks to at least one medication.^48^ Concordant results were reported in the RIGHT (Right Drug, Right Dose, Right Time Using Genomic Data to Individualize Treatment) protocol, created by the Mayo clinic/eMERGE initiative, which performed sequencing of a panel that included solute carrier organic anion transporter family member 1B1 (SLCO1B1), CYP2C19, CYP2C9, VKORC1 and CYP2D6. The study demonstrated that 99% of 1,013 individuals had at least one variant associated with increased risks to a medication.^49^

In the United States, medications with recommendations related to PGx constitute 18% of all prescriptions^50^ and 30% of the most prescribed medications that have a high PGx risk represent 738 million prescriptions a year.^51^ These data corroborate the idea of a positive impact of the preemptive PGx test, not only for the increase in therapeutic efficacy and cost-benefit, but also for the potential in preventing ADEs. In addition, a Dutch study showed a beneficial effect by reducing the risk of fluoropyrimidine-induced toxicity from 73% to 28% by genotype-guided dosing and reduction of drug-induced death from 10% to 0%.^52^

### Importance of Pharmacogenomics

Over the last years, PGx has emerged as an area of increasing interest and enthusiasm, as it essentially leads with the so-called “personalized medicine”, considering the influence of patients’ genomic variation on drug responses.^53^

Many benefits can be achieved with deployment of PGx:

- To increase therapeutic power and reduce likelihood of intoxication;
- To initiate a therapy in more appropriate time.

In addition, PGx may contribute to the reduction of costs in healthcare, as presented in Figure 1. It is important to mention that a large amount of deaths per year are related to ADEs, with a cost of approximately €80 billion.^54^

**Figure 1:** Advantages of pharmacogenomics and potential health cost reduction. *Due to possible earlier detection.

It is estimated that a considerable proportion of patients do not show a satisfactory response to drug treatment.^55^^,^^56^ In this regard, the FDA recommends, for example, that PGx tests be carried out before chemotherapy with mercaptopurine (a drug commonly used in the treatment of patients with acute leukemia) is initiated.^57^ This recommendation is based on the fact that, since the drug may cause severe side effects and increase the risk of infection, depending on individual’s genetic variant, the therapy may not achieve intended results.

The Genomics and Targeted Therapy Group, an arm of the FDA’s Office of Clinical Pharmacology, works to ensure that PGx strategies are applied appropriately by means of its functions of regulatory review, research, policy development, and professional education. Part of this work included the construction of a table describing pharmacological instructions of 161 drugs that display PGx information on the label. The last update provides a remarkable number of biomarkers associated to drugs used in several areas of medicine, many of them widely used in clinical practice^58^ (Table 1).

**Table 1:** Number of pharmacogenomic biomarkers in drug labeling by medical field, based on the US Food and Drug Administration table

Area	Biomarkers
Oncology	167
Infectious disease	35
Psychiatry	34
Neurology	29
Hematology	25
Anesthesiology	23
Cardiology	22
Gastroenterology	17
Rheumatology	11
Pneumology	10
Endocrinology	7
Inborn errors of metabolism	7
Urology	5
Dermatology	4
Toxicology	2
Transplant	1

Finally, it is expected that PGx soon become more accessible and that its responsible use contribute to more accurate drug prescription, with a higher likelihood of therapeutic success and a lower risk of ADEs.

### Association Between Genetic Variants and Drug Responses in Cardiovascular Diseases

It is widely known that factors like age, comorbidities, weight, and demographic aspects can contribute to significant differences in the response to a certain medication, as well as to the development of ADE.^59^^,^^60^ In this context, genetic variation may represent a cornerstone in this outcome. It is believed that many deaths could be prevented if physicians were aware of PGx profile of patients and prescribe them medications at correct doses.^61^ Patients diagnosed with the same disease are treated following the same therapeutic protocol although their responses to drug treatment may vary significantly. A tailored therapy can reduce ADEs and increase efficacy rates, as described in Figure 2. For example, there is a huge variation (up to 20 times) in the daily dose of one of the most used anticoagulants in clinical practice, warfarin, among patients.^62^ The dose of propranolol, a medication of the beta blocker class, may vary up to 40 times among users.^60^ Some medications widely used in cardiology, that may have important genetic associations, are listed in Table 2.

**Figure 2:** Potential clinical applications of Pharmacogenomics; Adapted from Johnson, 2003.^99^

**Table 2:** Association between genes and medications

Genes	Medications	Class	Genetic variant	Allele effect
*CYP2C9, VKORC1*	Warfarin	Vitamin K antagonist	CYP2C9*2 (p.Arg144Cys; rs1799853)	Reduced drug clearance; reduced dose required
			CYP2C9*3 (p.Ile359Leu; rs1057910)	Reduced drug clearance; reduced dose required
			VKORC1 (-1639G>A; rs9923231)	↑ Sensitivity to medication; Reduced dose required
*CYP2C19*	Clopidogrel	Receptor P2Y_12_ inhibitor	CYP2C19*2 (c.681G>A; rs4244285)	↑ Risk of cardiovascular events; loss of function; lower antiplatelet effect.
			CYP2C19*17 (c.-806C>T; rs12248560)	↑ Sensitivity to medication; loss of function; ↑ risk of bleeding
*SLCO1B1*	Simvastatin	HMG-CoA reductase inhibitor	SLCO1B1*5 (p.Val174Ala; rs4149056)	↑ Risk of myopathy or rhabdomyolysis
*ADRB1*	Atenolol, metoprolol	Beta blocker	ADRB1 (p.Ser49Gly; rs1801252)	Better blood pressure control; ↑ LVEF
			ADRB1 (p.Arg389Gly; rs1801253)	
*CES1*	Dabigatran	Direct oral anticoagulant	CES1 (G143E, rs71647871)	↑ Metabolism of medication and its metabolites
*ITGB3*	Aspirin	Antiplatelet agent	ITGB3 (Pl^A1/A2^ [T1565→C], rs5918)	↓ Antiplatelet effect

LVEF: left ventricular ejection fraction; CYP2C9: Cytochrome P450 2C9; VKORC1: vitamin K epoxide reductase C1; CYP2C19: P450 2C19; SLCO1B1: Solute carrier organic anion transporter family member 1B1; CYP4F2: cytochrome P450 family 4 subfamily F member 2; ADRB1: beta-1 adrenergic receptor; CYP11B2: Cytochrome P450 family 11 subfamily B member 2; FUT4: Fucosyltransferase 4; CES1: carboxylesterase 1; ITGB3: Integrin beta-3.

#### Warfarin

Warfarin is a vitamin K antagonist that has been largely used for the prevention of thrombotic events.^63^ Evidence has suggested that the individual response to warfarin and to other vitamin K antagonists, may be influenced by genetic variations in cytochrome P450 2C9 (CYP2C9) and vitamin K epoxide reductase C1 (VKORC1), target of these drugs,^64^^,^^65^ and polymorphisms of cytochrome P450 family 4 subfamily F member 2 (CYP4F2).^66^ Variations in the CYP2C9*2 and CYP2C9*3 have been shown to reduce enzymatic activity of CYP2C9 and inhibit the anticoagulant metabolism,^67^ whereas the polymorphism of VKORC1- 1639G>A seems to influence the pharmacodynamic response to vitamin K antagonists.^68^ For these issues, the FDA indicated the need for displaying PGx information in the package insert of warfarin.

In practice, heterozygous carriers for CYP2C9*2 or CYP2C9*3 may require a reduced warfarin dose, by approximately 30% and 47%, respectively, and homozygous carriers for CYP2C9*3 may require greater reductions (~80%).^69^^-^^71^ The -1639 G>A variant of the VKORC1 gene seems to reduce the expression of proteins, which in theory represents the requirement of a lower maintenance dose of warfarin compared with non-carriers of the variant.^72^ Also, combinations of some variants associated with ultrarapid metabolism limit the systematic definition of a therapeutic INR of these patients.^73^ In this scenario, the CPIC guidelines recommend considering a direct oral anticoagulant (e.g. edoxaban).^74^

#### Clopidogrel

In the United States, it is estimated that more than three million individuals are prescribed clopidogrel after stent implantation.^75^ Clopidogrel belongs to the class of thienopyridines and exerts antiplatelet effect.^76^ The individual response to clopidogrel may be altered by the polymorphism of CYP2C19.^77^

The CYP2C19*2 loss-of-function variant allele was associated with an increased risk of adverse cardiovascular events, including stent thrombosis during clopidogrel therapy.^78^ More specifically, the CYP2C19*2 (rs4244285) allele causes loss of function and was associated with reduced antiplatelet effect of the drug.^79^ Also, carriers of the CYP2C19 *3 (rs4986893) allele show poor response to clopidogrel and higher rates of recurrent adverse cardiovascular events as compared with non-carriers.^80^^,^^81^ It is important to mention that the frequencies of CYP2C19 *2 and CYP2C19 *3 are higher in Asian populations, suggesting that these individuals are more likely to be resistant to this medication.^82^ In contrast, the CYP2C19*17 (rs3758581) allele promotes gain of function and has been associated with increased enzymatic activity and improved platelet inhibition. Carriers of the CYP2C19*17 variant have been called ultrarapid metabolizers.^83^

In addition, race seems to play an important role in this scenario. Cresci et al.^84^ compared the effect of polymorphism of CYP2C19 on cardiovascular adverse events between patients with acute myocardial infarction in Caucasians and African Americans treated with clopidogrel. The authors found a significant association of the CYP2C19*2 allele with increased one-year mortality and an increasing trend in the incidence of recurrent myocardial infarction in Caucasians. The CYP2C19*17 was associated with higher one-year mortality and higher risk of bleeding in African Americans. Also, it is of note that CYP2C19*2 carriers that undergo percutaneous coronary intervention may have higher risk of stent thrombosis. In a RCT in which nearly 2,500 patients that were pre-treated with 600mg clopidogrel, there was a significanly higher stent thrombosis rate amongst CYP2C19*2 allele carriers, in 30 days, when compared with wild-type CYP2C19 allele carriers.^85^ In this same line, the meta-analysis conducted by Mega et al.,^86^ including studies on more severely ill patients receiving more aggressive treatment, found an increased risk of stent thrombosis when the allele *2 was identified by PGx.

Despite these evidences, a systematic review and meta-analysis including 15 studies did not corroborate these findings and did not show a clear influence of polymorphisms of the CYP2C19 gene on the clinical efficacy of clopidogrel,^87^ suggesting that the use of individualized antiplatelet regimens guided by CYP2C19 genotype is not justified.

Today, the American College of Cardiology, in conjunction with the American Heart Association, does not recommend PGx tests for CYP2C19 as a routine approach.^88^ However, a more recent meta-analysis demonstrated that patients who may benefit from the PGx study are those with coronary artery disease, undergoing percutaneous myocardial revascularization.^89^ In this context, the CPIC officially recommends that patients with acute coronary syndrome and even those undergoing percutaneous coronary intervention undergo PGx testing. The CPIC emphasizes that those patients with one or two copies of loss-of-function variants should receive alternative antiplatelet agents (prasugrel or ticagrelor), to reduce the risk of adverse cardiovascular events.^90^ On the other hand, PGx testing is not indicated for other patient populations (e.g. atrial fibrillation patients), in which the use of clopidogrel is debatable.

Claassens et al.^91^ conducted a recent RCT to evaluate the results of a genotype-guided antiplatelet therapy in patients with acute myocardial infarction with ST segment elevation. Patients were assigned to receive either clopidogrel, based on early CYP2C19 genetic testing (non-carriers of loss-of-function alleles received clopidogrel) or standard treatment with either ticagrelor or prasugrel. No difference was found between the groups in the incidence of thrombotic events and therefore, the genotype-guided strategy was noninferior to standard treatment with ticagrelor or prasugrel, which is more expensive and associated with higher incidence of bleeding.^91^

Results of the TAILOR PCI clinical trial were recently published. The authors assessed a genotype-guided strategy (n=2,652) versus standard therapy (n=2,650) in patients with stable or unstable coronary artery disease, aiming to determine whether genetic testing could identify the best anti-platelet therapy in these individuals. In the genotype-guided group, CYP2C19 *2 or *3 carriers received ticagrelor 90 mg twice a day or non-carriers received clopidogrel 75 mg daily. In the standard therapy group, patients received clopidogrel 75 mg daily and underwent genotyping test at 12 months. The primary outcome was cardiovascular death, myocardial infarction, stroke, stent thrombosis or recurrent ischemia in 12 months. The primary outcome and the incidence of bleeding were not different between the treatment groups. However, it is worth mentioning the 34% reduction in these events at one year and a 40% reduction in the number of events per patient in the genotype-guided group. Finally, a post-hoc analysis revealed a reduction of approximately 80% in the adverse event rate in the first three months of treatment in the group of patients randomized to the genotype-guided therapy arm.^92^^,^^93^

#### Beta Blockers

This class of medications has been extensively used in the treatment of cardiac arrhythmias, chest pain, myocardial infarction, and hypertension.^94^ The genes associated with individual response to beta blockers include the CYP2D6, the beta-1 adrenergic receptor (ADRB1), the beta-2 adrenergic receptor (ADRB2) and G protein-coupled receptor kinase 5 (GRK5).^95^ For example, some beta-blockers, including propranolol and metoprolol, are metabolized by the CYP2D6, which very frequently presents with a ‘loss of funtion’ variant.^34^ Evidence has suggested that hypertensive patients, homozygous for the wild-type allelic variant Arg389, showed a 3-fold greater reduction in diurnal diastolic pressure with metoprolol compared with carriers of the allelic variant Gly389.^96^ Other findings,^97^ despite not so consistent, indicated that patients homozygous for the ADBR1 Arg389 haplotype seem to present a more satisfactory response to the family of beta-blockers, with a better left ventricular ejection fraction as compared with carriers of the Gly389 allele.^97^

Regarding the skin color, the higher frequency of the Gly389 allele in African Americans compared with white skin may be a plausible explanation for the reduced response to beta blockers. Although ethnicity and polymorphisms of ADRB1 have been reported as independent predictors of responses to beta blockers,^98^ further prospective studies to elucidate the role of these genetic variants in ethnicity-specific responses are needed.

Therefore, there are no recommendations for the use of PGx in guiding the use of beta blockers in heart failure treatment.

#### Statins

Statins represent a class of medications that target the HMG-CoA reductase (3-hydroxy-3-methyl-glutaryl-coenzyme A reductase) inhibition, and are used to reduce cholesterol levels, especially LDL cholesterol.^99^ Combined with changes in lifestyle, these drugs are considered first-line therapy for primary and especially secondary prevention of CVDs. However, large interindividual variability has been observed in the extension of LDL reduction, explained, in part, by environmental and genomic factors.^100^ Thus, a dose tailoring may be needed for each patient, to obtain a more effective response.

More recently, Licito et al.^101^ evaluated association of the PGx profile and neuromuscular pain in 76 type 2 diabetes mellitus patients and previous CVD using anti-diabetic and anti-cholesterolemic agents, such as statin. Different variants were studied, including the SLCO1B1, ABCB1, ABCC8, and drug biotransformers of cytochrome P450 Family (CYP) including CYP2C9*2 CYP2C9*3 CYP2C8*3, and CYP3A4*22. Approximately 17% of 35 patients treated with statin had neuromuscular pain. The PGx analysis showed a lack of any correlation between candidate gene polymorphisms and toxicity, except for the SLCO1B1 T521C allele. Thus, when available, analysis of the SLCO1B1 T521C variant is suggested, to enable clinicians to optimize the therapy prescribed, aiming at minimizing neuromuscular pain and maximizing the benefits from statins.

Also, the most strongly associated variant (with SLCO1B1), c.521T>C, reduces SLCO1B1 transport function, which can affect statin clearance, resulting in increased risk for toxicity in skeletal muscle. A meta-analysis of nine case-control studies, involving 4,500 patients, showed that individuals with the variant allele C were likely to experience statin-related myopathy (CT + CC versus TT: odds ratio = 2.09; 95%CI = 1.27-3.43).^102^

### Possible Barriers to the Implementation of Pharmacogenomics

Due to the advances in technology and sequencing techniques, the costs of PGx analysis have drastically reduced in the last years (Moore’s law), facilitating its use in clinical practice. However, the relatively high cost of PGx tests represents a barrier to its wider implementation. Also, there is a lack of familiarity by healthcare providers, a lack of a platform standardizing investigation and academic thinking and an insufficient volume of studies demonstrating the benefits of PGx. Those are factors that contribute to a low acceptance.

Nevertheless, there is currently a global effort to overcome these obstacles, including the development of large studies,^103^ like the UK’s 100.000 Genomes Project,^104^ the PREemptive PGx testing for prevention of Adverse drug REactions (PREPARE), with participation of seven European countries.^105^ On the other side of the North Atlantic, in the United States, the Electronic Medical Records and Genomics (eMERGE),^106^ the Network and the Implementation of Genomics in Practice (IGNITE),^107^ and the Clinical Sequencing Evidence Generating Research Consortium,^108^ are part of a series of projects funded by the National Human Genome Research Institute, with an estimated investment of at least U$ 775 million in genetic research from 2007 to 2022. In Asia, the South East Asian PGxs Research Network (SEAPharm) is a collaborative effort of five Asian countries to develop studies in PGx.^109^ In general, these studies are aimed to define, provide and analyze evidence of the clinical usefulness of genomic sequencing to guide treatment, cost-efficacy and the costs of its broad implementation in clinical practice. Of the available results, a review including 44 cost-benefit analyzes showed that 30% of them showed cost-effectiveness and 27% even showed a cost reduction.^110^ This may lead to an optimistic perspective of the future of PGx.

Among global initiatives’ many contributions for the expansion of PGx in clinical practice, some of the crucial aspects worth to highlight are: education and training of healthcare providers, investment in technology, in research and in healthcare centers’ structure. Projects involving PGx not only contribute to knowledge in the area, but also promote training of healthcare professionals to an adequate practice of genomic medicine, which requires the adoption of basic routines that may be less relevant to other medical specialties. For example, the involvement of family members in counseling and treatment planning, and confidentiality that ensures that genetic information is being used exclusively for the purpose of assistance (thereby avoiding the inappropriate use of PGx in relation to legislation, insurance, marketing or employment relationship). Education and training play a central role for the acceptance of genomic medicine in clinical practice, and acceptance, in turn, is the key to. The effort for data generation is of significant help, but commitment and involvement of healthcare providers that seek excellence are essential. The scale and the progress of efforts and investments in the world make clear the importance and potential attributed to genomic medicine, as it may be considered a component of high-quality medicine and one of the cornerstones of precision medicine.

### Final Considerations

1) Area of consensus: PGx tests may be helpful in the optimization of drug treatments, allowing greater pharmacological safety;
2) Area of controversy: whether PGx tests may be extensively applied, including for the prescription of medications whose benefits are not so clear;
3) Area of expansion: individual genotype data have become more and more available to consumers. This will probably increase the demand for a personalized prescription, indicating that prescribers should consider PGx data. For example, we can site the 100,000 Genomes Project. This amazing project will provide complete genome sequences that may someday be included in patients’ medical records. This seems to be a valuable information in personalized prescription.
4) Promising research areas: development of novel drugs targeting specific genetic risk factors of diseases. These medications could be prescribed for individuals with a high-risk genotype. It is likely that PGx information become routinely available in the future, mainly in technologically advanced environments. This could influence the prescription of several drugs other than those requiring PGx tests prior to prescription.
